# Detecting Alpha Synuclein Seeding Activity in Formaldehyde-Fixed MSA Patient Tissue by PMCA

**DOI:** 10.1007/s12035-018-1007-y

**Published:** 2018-03-27

**Authors:** Katelyn Becker, Xinhe Wang, Kayla Vander Stel, Yaping Chu, Jeffrey Kordower, Jiyan Ma

**Affiliations:** 1 Center for Neurodegenerative Science, Van Andel Research Institute, 333 Bostwick Avenue N.E, Grand Rapids, MI 49503 USA; 2 Department of Neurological Sciences, Rush University Medical Center, Chicago, IL 60612 USA

**Keywords:** Alpha synuclein, Amyloid, Protein misfolding cyclic amplification, Parkinson’s disease, Multiple system atrophy, Synucleinopathies

## Abstract

Alpha synuclein (α-syn) is central to the pathogenesis of a group of neurodegenerative disorders known as synucleinopathies, including Parkinson’s disease (PD), dementia with Lewy bodies (DLB), and multiple system atrophy (MSA). Aggregation of α-syn is the pathologic hallmark of these disorders and is intimately associated with the pathogenic changes. The prion-like hypothesis postulates that the aggregated α-syn provides a template to seed the aggregation of normal α-syn and spread the pathology. Thus far, it remains unclear whether aggregated α-syn can be a useful biomarker for diagnosis and/or tracking disease progression, which is mainly due to the lack of a suitable biochemical assay. The protein misfolding cyclic amplification (PMCA) technique is known for its enormous amplification power to detect the seeding activity of protein aggregates such as prions. In this study, we adapted PMCA for detecting the seeding activity of α-syn. By extensively optimizing the PMCA parameters, we developed a protocol that is able to sensitively and quantitatively detect the seeding activity of as little as 100 attomoles (10^−16^ mol) of α-syn aggregate. Using our protocol, we detected α-syn seeding activity from a histologically positive, formaldehyde-fixed MSA sample, but not with the histologically negative, formaldehyde-fixed control sample. Our results confirmed that the α-syn in MSA patient’s brain does contain seeding activity, which remains active even after fixation. Moreover, we also established that PMCA with sonication is a sensitive and quantitative method for detecting α-syn seeding activity, which can be further adapted to more accessible patients’ samples to evaluate α-syn aggregates as a biomarker for synucleinopathies.

**Electronic supplementary material:**

The online version of this article (10.1007/s12035-018-1007-y) contains supplementary material, which is available to authorized users.

## Introduction

Parkinson’s disease (PD) is the second most common neurodegenerative disorder characterized by the degeneration of dopaminergic neurons in the substantia nigra and the aggregation of alpha synuclein (α-syn) protein in the form of Lewy bodies or Lewy neurites [1–4]. The association between aggregated α-syn and the pathogenic changes in PD is well supported [5–8], particularly with the identification of familial PD caused by α-syn mutations or multiplications of the gene encoding α-syn [9–12], and the discovery of sporadic PD risk alleles in the α-syn gene by genome-wide association study [13, 14]. Besides PD, α-Syn aggregates are also present in other neurodegenerative disorders including dementia with Lewy bodies (DLB) and multiple system atrophy (MSA) [5, 15]; these diseases are classified as synucleinopathies [16]. Currently, there is no biochemical test available to diagnose or monitor the progression of PD or other synucleinopathies [17], which is a major barrier to developing effective therapies for these devastating diseases.

Aggregated α-syn has been proposed as a biomarker for disease progression because of its close association with neurodegeneration in synucleinopathies [18, 19]. Braak and colleagues revealed that the staging of α-syn aggregates correlates with the progression of PD [20, 21]. However, the histological analysis of α-syn aggregates used to establish Braak staging, which is also the post-mortem analysis used to confirm diagnosis, does not meet the requirement for a rapid, sensitive, and pre-mortem biomarker useful for diagnosis and tracking of disease progression. To achieve these goals, the development of sensitive imaging or biochemical assays of accessible tissue samples is crucially needed [19].

In diseased brains, α-syn aggregates exist in the form of amyloid fibrils, which are well known for their seeding ability. That is, the amyloid fibrils can act as templates for the conformational change of normal α-syn, resulting in the growth of the fibrils [18]. This seeding ability of α-syn aggregates has been demonstrated by injecting patients’ brain homogenates into mice or primates, which resulted in disease-relevant aggregates composed of host-encoded α-syn [22, 23]. These findings support the prion-like hypothesis [21], which posits that in diseased individuals, α-syn aggregates spread in a manner similar to that of prions, leading to the widespread pathology seen in synucleinopathies. However, whether the prion-like spread of α-syn is the underlying pathogenic mechanism for synucleinopathies is still debated [24].

The potent seeding ability of prions led to the development of sensitive biochemical assays to detect the pathogenic prion protein (PrP) isoform in humans and animals [25–28]. Among those assays, the protein misfolding cyclic amplification (PMCA) assay and the real-time quaking-induced conversion (RT-QuIC) assay have achieved exceptional sensitivity and specificity. When tissues or body fluids containing prion seeds are mixed with normal brain homogenates and subjected to successive cycles of sonication and incubation, the PMCA assay propagates the pathogenic PrP conformation and prion infectivity simultaneously [29]. It is highly sensitive and able to detect prions in blood from presymptomatic and symptomatic patients with variant Creutzfeldt-Jakob disease with 100% sensitivity and specificity [28]. The RT-QuIC assay detects prion seeding by intermittent shaking of prion-containing tissues or body fluids mixed with recombinant PrP and thioflavin T (ThT) [26]. Because ThT displays an enhanced fluorescence signal when it binds to β-sheet-rich structures like amyloid fibrils [30], the RT-QuIC assay allows a real-time monitoring of prion-seeded fibril growth [26]. The application of RT-QuIC to diagnose human Creutzfeldt-Jakob disease has achieved over 97% sensitivity and 100% specificity with nasal brushings of the olfactory mucosa [25, 31].

Recently, several groups have reported their attempts to use these assays to detect α-syn aggregates in tissue lysates or cerebrospinal fluid (CSF). Using preformed recombinant α-syn fibrils as seed, Herva et al. reported that α-syn PMCA reproducibly amplified α-syn amyloid fibrils and the amplification was inhibited by known anti-amyloid compounds [32]. Jung et al. amplified α-syn aggregates from diseased tissues of transgenic mouse lines [33]. Using RT-QuIC, Sano et al. detected the prion-like seeding of misfolded α-syn in the fresh brain homogenates of DLB patients [34]. For attempts to use these assays for the diagnostic purpose, Fairfoul et al. adapted the RT-QuIC assay to detect α-syn aggregates in the CSF of a small group of patients suffering from various synucleinopathies and showed high sensitivity and specificity [35]. Shahnawaz et al. applied a modified PMCA protocol—cycles of 1 min shaking followed by 29 min incubation, but without sonication—to patients’ CSF and achieved an overall sensitivity of 88.5% and a specificity of 96.9% [36].

A major challenge for developing a detection technique is the very low amount of α-syn aggregate in accessible tissue sources [37–40]. One goal of this study is to establish a reproducible and quantitative protocol with enhanced sensitivity, as this is crucial for diagnosis and for monitoring disease progression. Because the sonication step usually stimulates more-robust amyloid fibril growth from a small amount of seed [41], we chose to optimize the classical PMCA protocol with the sonication step. Our optimized protocol was able to quantitatively detect α-syn aggregates down to 100 attomoles. Using this protocol, we successfully detected α-syn seeding activity in formaldehyde-fixed brain tissue from an MSA patient, demonstrating the sensitivity of this assay and revealing the powerful seeding activity of α-syn aggregates in MSA.

## Methods

### Purification of Recombinant α-Syn

Recombinant α-syn was purified according to the method published previously [42]. Briefly, a single colony of BL21 *E. coli* transformed with human α-syn-expressing plasmid was grown until the optical density at 600 nm reached 0.6 to 0.9; cells were then induced with isopropyl β-D-1-thiogalactopyranoside (IPTG) for 4 h. For purification, cells in a high-salt buffer (750 mM NaCl, 10 mM Tris, 1 mM EDTA, and 1 mM phenylmethylsulfonyl fluoride) were lysed by sonication and boiling. After removal of cell debris, the lysate was dialyzed overnight in 10 mM Tris, 50 mM NaCl, and 1 mM EDTA and then loaded onto a Superdex 200 column (GE Healthcare Life Sciences). Fractions containing α-syn were identified by SDS-PAGE and Coomassie blue staining, and dialyzed overnight in 10 mM Tris, 25 mM NaCl, and 1 mM EDTA. Protein was then subjected to chromatographic separation on a Hi-Trap Q HP anion-exchange column (GE Healthcare Life Sciences) and eluted with a 0 to 1 M NaCl gradient, where α-syn elutes around 350 mM NaCl observed by absorption at 280 nm. The purity of α-syn was confirmed by SDS-PAGE and Coomassie blue staining. Purified α-syn was dialyzed into 10 mM Tris, divided into aliquots, and stored at − 80 °C until use.

### PMCA Reaction

Upon thawing, purified α-syn was centrifuged at 100,000×*g* for 30 min at 4 °C to remove any aggregates that may have formed during the freeze-thaw process. Monomeric α-syn was then diluted to 50 μM (0.723 mg/ml) in 10 mM Tris pH 7.5, 150 mM NaCl in 0.2 mL PCR tubes (GeneMate) with about 10 zirconium oxide beads (1 mm diameter; Next Advance). PCR tubes were then placed in a Q700 sonicator (Qsonica) connected to a circulating water bath. Unless specified, the PMCA was carried out with repeated cycles of 10 s of sonication and 29 min 50 s of incubation at 37 °C. At various time points, 2 μL of sample was removed and was incubated with 198 μL thioflavin T solution (20 μM ThT, 50 mM glycine, pH 8.5) in a 96-well plate (Greiner Bio-One) for 5 min at room temperature. ThT readings were performed on a Tecan M200 plate reader with an optimized gain of 100 (excitation at 440 nm and emission at 480 nm). For seeded reactions, α-syn fibrils formed by PMCA were centrifuged and the pellet was resuspended in 10 mM Tris buffer, pH 7.5 to 10× of the desired final concentration. Seed was added to the PMCA reactions at a 1:10 ratio.

### Electron Microscopy

Hydrocarbon contamination was removed from mesh carbon grids pre-coated with copper (Electron Microscopy Sciences) using the Solarus Advanced Plasma Cleaning system model 950 (GATAN). Grids were then incubated with 3 μL of either α-syn fibrils or monomers for 30 s. Samples were stained by a quick rinse with one drop of 1% uranyl acetate, followed by a 30-s incubation with a second drop of 1% uranyl acetate. Samples were analyzed and imaged using a Tecnai G2 Spirit TWIN transmission electron microscope.

### Primary Cortical Neuron Culture

P1 mouse cortical neurons were isolated and cultured according to a published protocol [43]. At 7 days in vitro (DIV), cells were treated with preformed α-syn fibrils at 70 nM. For immunofluorescence staining, cells at 16 DIV were fixed with 4% formaldehyde in culture medium at 37 °C for 15 min and then permeabilized with 0.2% Triton-100 for 10 min at room temperature. Anti-MAP2 (Sigma, 1:500) and anti-α-syn phospho (Ser129) (Abcam, 1:1000) were used as the primary antibodies and Alexa 488-conjugated goat anti-mouse IgG and Alexa 594-conjugated goat anti-rabbit IgG (ThermoFisher) as the secondary antibodies. Images were visualized with an Olympus IX83 microscope.

### Preparation of Mouse Brain Homogenate

A wild-type C57BL mouse was sacrificed according to the IACUC-approved protocol at the Van Andel Research Institute. The brain was isolated, homogenized at 1:10 (weight/volume) in 10 mM Tris pH 7.5, aliquoted, and stored at − 80 °C. When needed, an aliquot was thawed and added directly to the PMCA reaction with a final concentration of 1%. For seeded reactions, seed was created as described above, serially diluted, and added to the brain homogenate at 10× the final concentration, and then added to the PMCA reaction.

### Preparation of Formaldehyde-Fixed Human Brain Homogenate

Human tissues were fixed in 4% paraformaldehyde at 4 °C for 5 days. After fixation, brain blocks were washed with PBS and then cryopreserved in 0.1 M PBS pH 7.4 containing 2% dimethyl sulphoxide, 10% glycerol for 2 days, followed by 2% dimethyl sulphoxide and 20% glycerol in PBS for at least 2 days. The blocks were kept in this solution at 4 °C until sectioning. The α-syn aggregates in the brain were confirmed by immunohistochemical staining with an antibody against serine 129-phosphorated α-syn (abcam, ab51253). The samples were homogenized with 0.5 mm glass beads (Next Advance) using a bullet blender (Next Advance) at 1:10 (weight/volume) in PBS. Homogenates were aliquoted and stored at − 80 °C. For PMCA reaction, a frozen aliquot was thawed, briefly sonicated for 1 min in the water bath sonicator, and then added at a final concentration of 1% to PMCA reactions.

### Statistical Analysis of Fibril Growth

Raw ThT readings were analyzed using the GraphPad Prism software version 6.05. Kinetic analysis of PMCA fibril growth was performed by fitting the data with a sigmoidal curve (displayed on each graph as a solid line). Lag phases were calculated as the time required for the ThT fluorescent signal to reach three times of the baseline reading. Statistical analysis was carried out using a one-way ANOVA; *p* value symbols are as follows: * ≤ 0.05, ** ≤ 0.01, *** ≤ 0.001, **** ≤ 0.0001. Lag phases plotted as a function of the logarithm of the seed amount were fitted with a semilog line.

## Results

### Quantitative Detection of α-Syn Fibrils by PMCA

It is well documented that recombinant α-syn is able to self-nucleate to form amyloid fibrils, a process that can be accelerated by agitation [44]. Similarly, the sonication step in PMCA also significantly accelerates recombinant α-syn self-nucleation [32]. To sensitively detect a small amount of α-syn aggregate, we would need to increase the gap between unseeded and seeded reactions. Thus, we focused on optimizing the PMCA conditions that would delay the growth of the unseeded reaction and/or enhance the growth of seeded reactions.

**Fig. 1:**
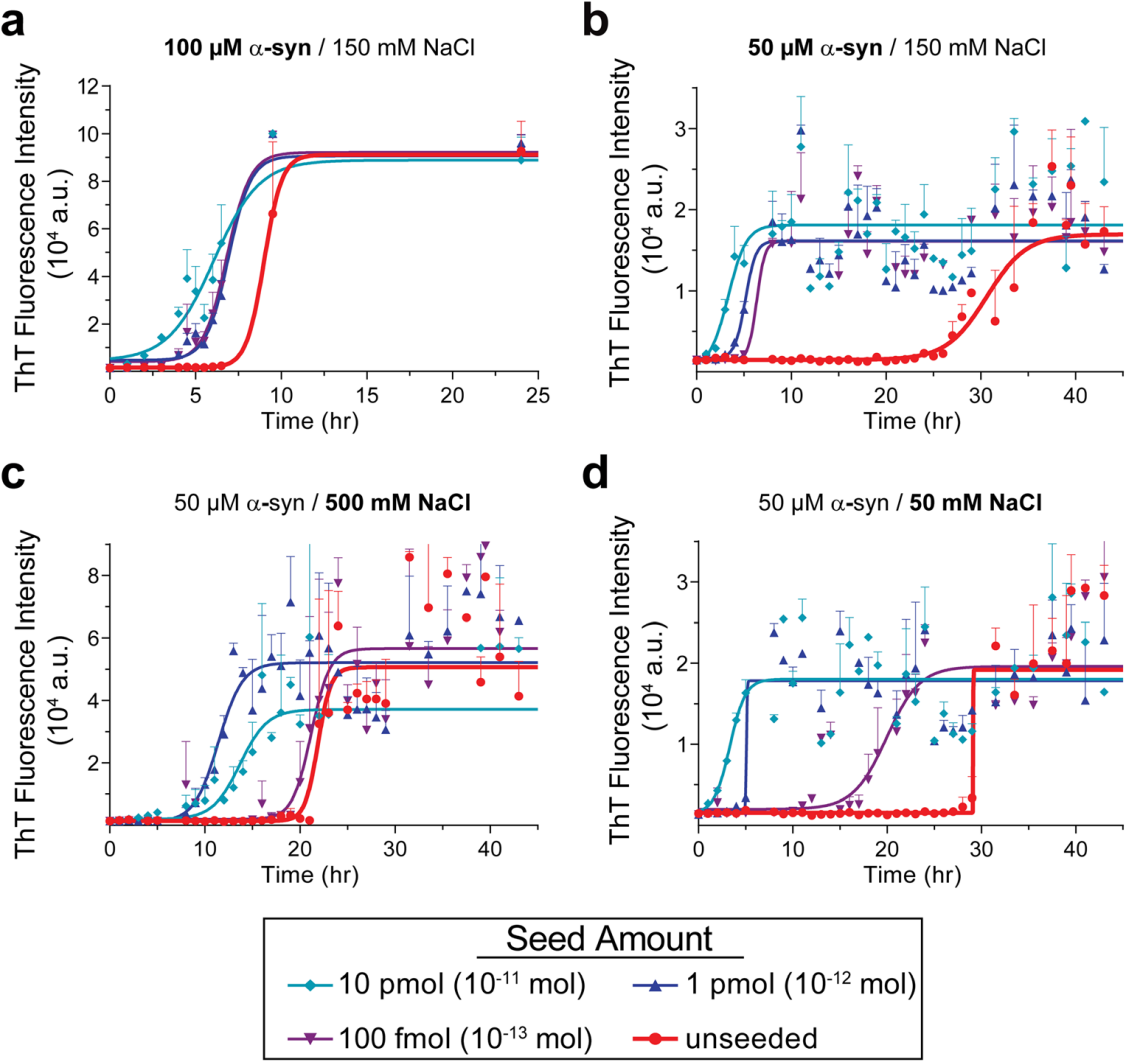
Optimizing the PMCA protocol. Growth kinetics of seeded and unseeded α-syn PMCA with high (100 μM, **a**) or low (50 μM, **b**) monomeric α-syn concentration, and with high (500 mM, **c**) or low (50 mM, **d**) NaCl concentration. Figures are representative graphs of three independent experiments; each point represents the mean ± SD of six replicates for each reaction except in **a**, which had three replicates. Assembly was monitored by ThT fluorescence intensity at 480 nm, with excitation of 440 nm. Data was fitted with a sigmoidal curve using the GraphPad Prism software

We started the PMCA reaction with 100 μM monomeric α-syn and 150 mM NaCl; at this concentration, α-syn spontaneously formed fibrils in 7 h (Fig. 1a). We tested various α-syn concentrations (Online Resource Fig. S1) and found that the PMCA reaction with 50 μM monomeric α-syn (Fig. 1b) delayed unseeded fibril growth to around 26 h with minimal loss in the ThT signal. Increasing the NaCl concentration to 500 mM (Fig. 1c) accelerated α-syn fibril formation, resulting in a shorter lag phase for the unseeded reaction. Decreasing the salt concentration to 50 mM NaCl (Fig. 1d) led to an overall delay in fibril growth and relative to reactions carried out with 150 mM NaCl, the seeding ability was significantly reduced as well (compare Fig. 1d to b). We also tested other parameters, including pH, temperature, incubation time, and sonication intensity, and our results showed that all of them affect the fibril growth (Online Resource Figs. S2-S4). During these analyses, we found that the intensity of the ThT fluorescence signal can be influenced by many factors, including batch-to-batch difference of recombinant α-syn, ThT concentration, temperature, salt concentration, different plate readers, etc. But the lag time of fiber growth is much more consistent and can be used as a measure for the seeding activity. Considering all these factors, we developed a PMCA protocol with optimized sensitivity and reproducibility (Online Resource Fig. S5).

**Fig. 2:**
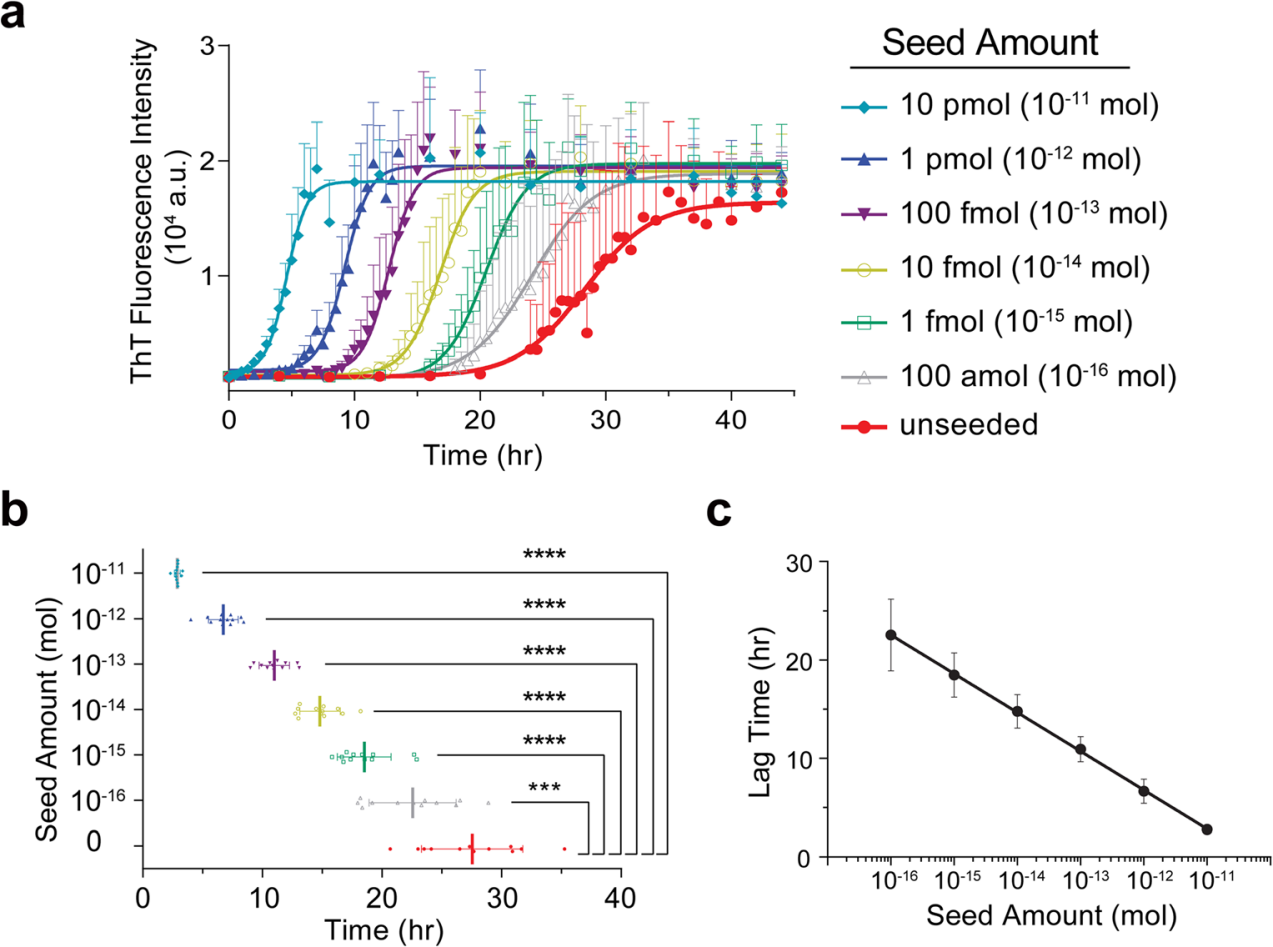
Quantitative analysis of seeded PMCA. **a** Detailed growth kinetics of seeded and unseeded α-syn reactions. Each point represents the mean ± SD of 12 replicates, and the data was fitted to a sigmoidal curve using GraphPad Prism. **b** Data points represent the calculated lag phases for each of the 12 replicates graphed according to the seed amount, with the mean (vertical line) and the SD (horizontal line) shown for each condition. Statistical significance between the lag phases was determined using a one-way ANOVA, and the statistical comparison depicted is the significance between each seed amount and the unseeded reaction. *P* value symbols are * ≤ 0.05, ** ≤ 0.01, *** ≤ 0.001, **** ≤ 0.0001. **c** The mean lag times are plotted as a function of the logarithm of seed amount added to the reaction and are fitted with semilog line

To determine the sensitivity of our optimized PMCA protocol, we used various amounts of preformed α-syn amyloid fibrils (PFFs) as seed (Fig. 2). The mean lag phase of the unseeded reaction was 27.56 h, with a standard deviation of 4.25 h (Fig. 2, red). When 10 pmol of seed was added to the reaction, the mean lag phase reduced to 2.79 h with a standard deviation of 0.25 h. Lower concentrations of seed resulted in longer lag times and larger standard deviations (Fig. 2b), indicating that the addition of seed helps to negate the variability that is often seen in α-syn fibril growth. More importantly, all of the lag times of the seeded reactions were significantly shorter than that of the unseeded reaction (Fig. 2b).

The seeded reactions were also all significantly different from each other (Supplemental Table). We were able to fit the mean lag times with a semilog line (*R*^2^ value of 0.9233) when plotted as a function of the logarithm of the seed amount added to PMCA (Fig. 2c), demonstrating the ability of this assay to quantify the amount of seed based on the lag time. Our optimized PMCA protocol was able to quantitatively detect the presence of α-syn aggregates to as low as 100 amol, which is 2 × 10^−8^% of the α-syn in the PMCA substrate. This detection level is significantly lower than that of previous α-syn PMCA protocols [32, 33]. The high sensitivity of this assay will be valuable for the detection of seeding-competent α-syn species in human samples.

### Characterization of PMCA Product

**Fig. 3:**
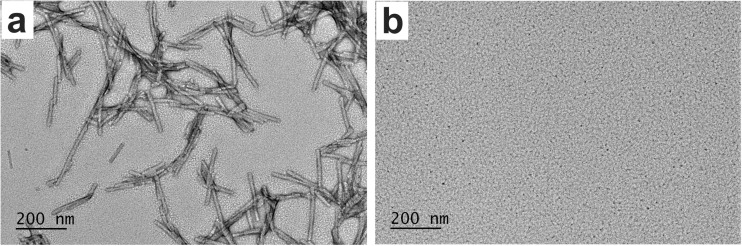
Images of α-syn PMCA products and α-syn monomers. **a** The α-syn amyloid fibrils generated by PMCA, and **b** the α-syn monomers imaged using TEM at 18,500× magnification and 120 kV. The scale bar represents 200 nm

The PMCA products were imaged via transmission electron microscopy (Fig. 3a), which showed a typical amyloid fibril morphology with an average width of the fibril about 13 nm. To determine whether recombinant α-syn PMCA products had the ability to seed endogenous α-syn, we adapted a recently published protocol of applying PFFs to seed aggregation of endogenous α-syn in primary neurons [43, 45]. Because mouse primary cortical neurons were used for this analysis and it is known that there is a transmission barrier between human and mouse α-syn [46], we used recombinant mouse α-syn to carry out the PMCA. The collected PMCA products were diluted to 7 μM, sonicated, and added to the mouse primary cortical neurons that had been cultured for 7 days; the neurons were then cultured for an additional 9 days.

**Fig. 4:**
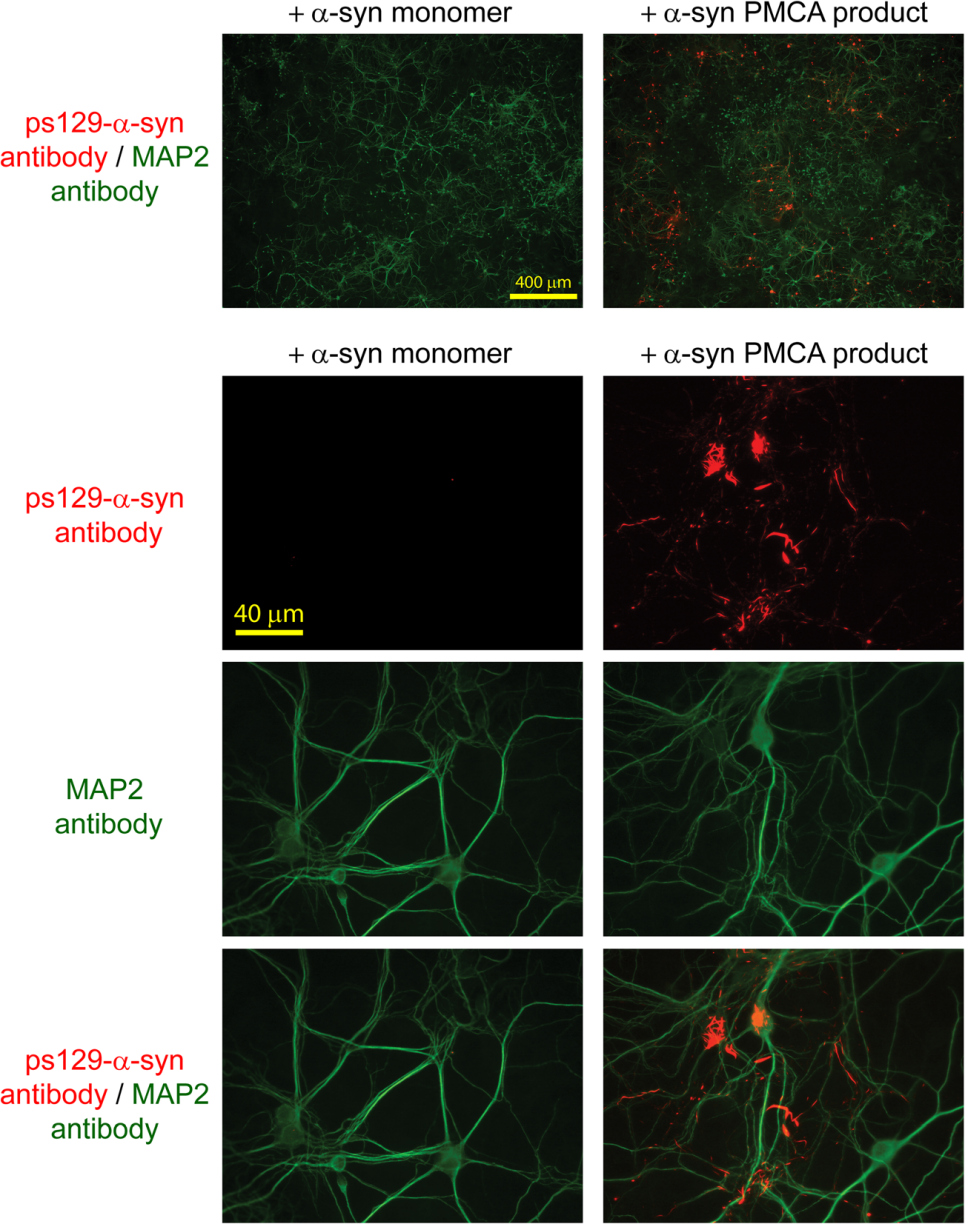
Mouse primary cortical neurons incubated with monomeric α-syn or PMCA product. Neurons at 7 DIV were incubated with monomeric α-syn (left) or PMCA product (right). Immunofluorescence staining was performed at 16 DIV. Fixed cells were stained for serine 129–phosphorylated α-syn (ps129-α-syn) (red) and MAP2 (green)

The aggregation status of endogenous α-syn was assessed by immunofluorescent staining with an antibody against serine 129-phosphorylated α-syn (ps129-α-syn), a well-documented marker for aggregated α-syn [42]. In contrast to primary neurons seeded with α-syn monomer, in which there was almost no ps129-α-syn staining (Fig. 4, left panels), a prominent ps129-α-syn signal was detected in primary neurons treated with PMCA products, and the majority of the signal was co-localized with the neuronal marker MAP2 (Fig. 4, right panels). This result reveals that the PMCA product maintains the capability to seed endogenous α-syn into an aggregated state.

### Detection of α-Syn Seed in the Presence of Mouse Brain Homogenate

**Fig. 5:**
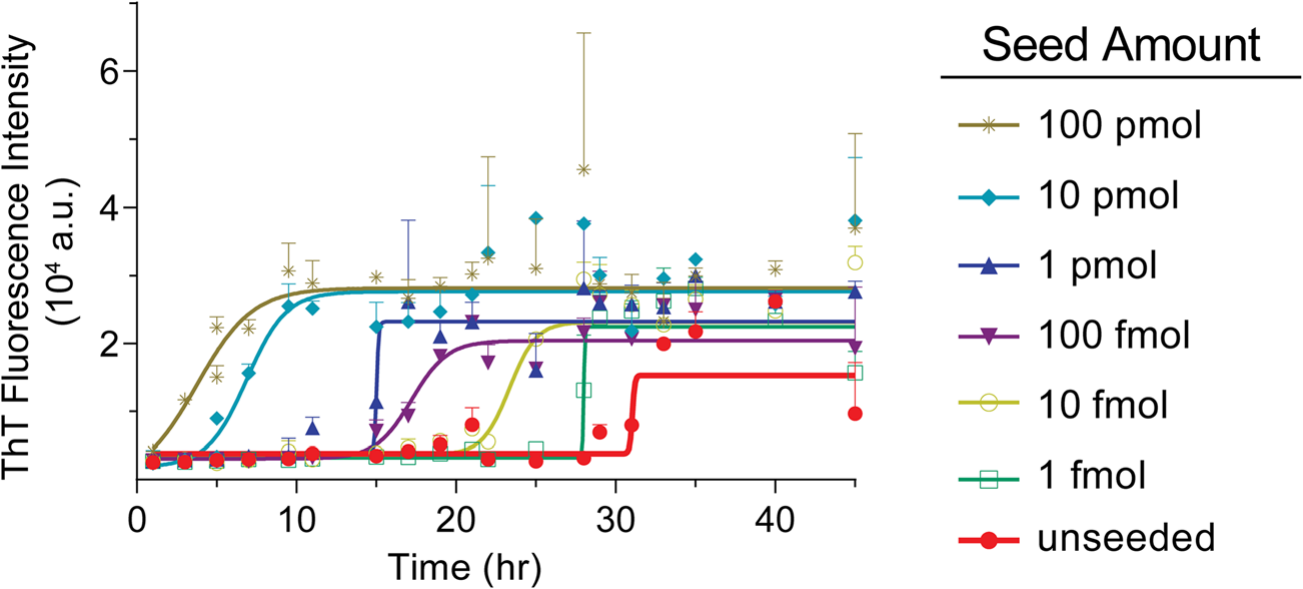
PMCA in the presence of mouse brain homogenates. Growth kinetics of seeded and unseeded α-syn after the addition of 1% (*w*/*v*) homogenized mouse brain tissue. The figure is a representative graph of three independent experiments; each point represents the mean ± SD (error bars) of six replicates for each reaction. Fluorescence measurement and data fitting were the same as in Fig. 1

Our goal is to detect α-syn seeding capability in biological specimens. Given that certain components in the specimens may interfere with α-syn fibril growth, we determined the sensitivity of our assay in the presence of mouse brain homogenates. The PMCA reaction was carried out in the presence of 1% (volume/volume) mouse brain homogenates, which were prepared by homogenizing mouse brain in 10 volumes of 10 mM Tris, pH 7.5 (weight/volume). The presence of the brain homogenate increased the lag times of all reactions by about 5 h, (compare Figs. 5 to 2a), suggesting that some component(s) of mouse brain homogenates delayed α-syn fibrilization. However, even with this delay, our protocol was still able to detect α-syn aggregates to as low as 1 fmol (Fig. 5). This finding confirmed that our protocol is able to detect small amounts of α-syn aggregates in biological samples.

### Detection of α-Syn Aggregates in Formaldehyde-Fixed Brain Tissue from an MSA Patient

Next, we tested the sensitivity of our assay on a real biological sample. Because it has been reported that α-syn seeding activity can be detected in formaldehyde-fixed tissue of aged Thy1-hA53Tα-syn transgenic mice by injecting brain lysates into young transgenic mice [47], we decided to test our biochemical assay with a formaldehyde-fixed MSA patient brain, which is a much greater challenge to the sensitivity of our assay than using other biological samples.

**Fig. 6:**
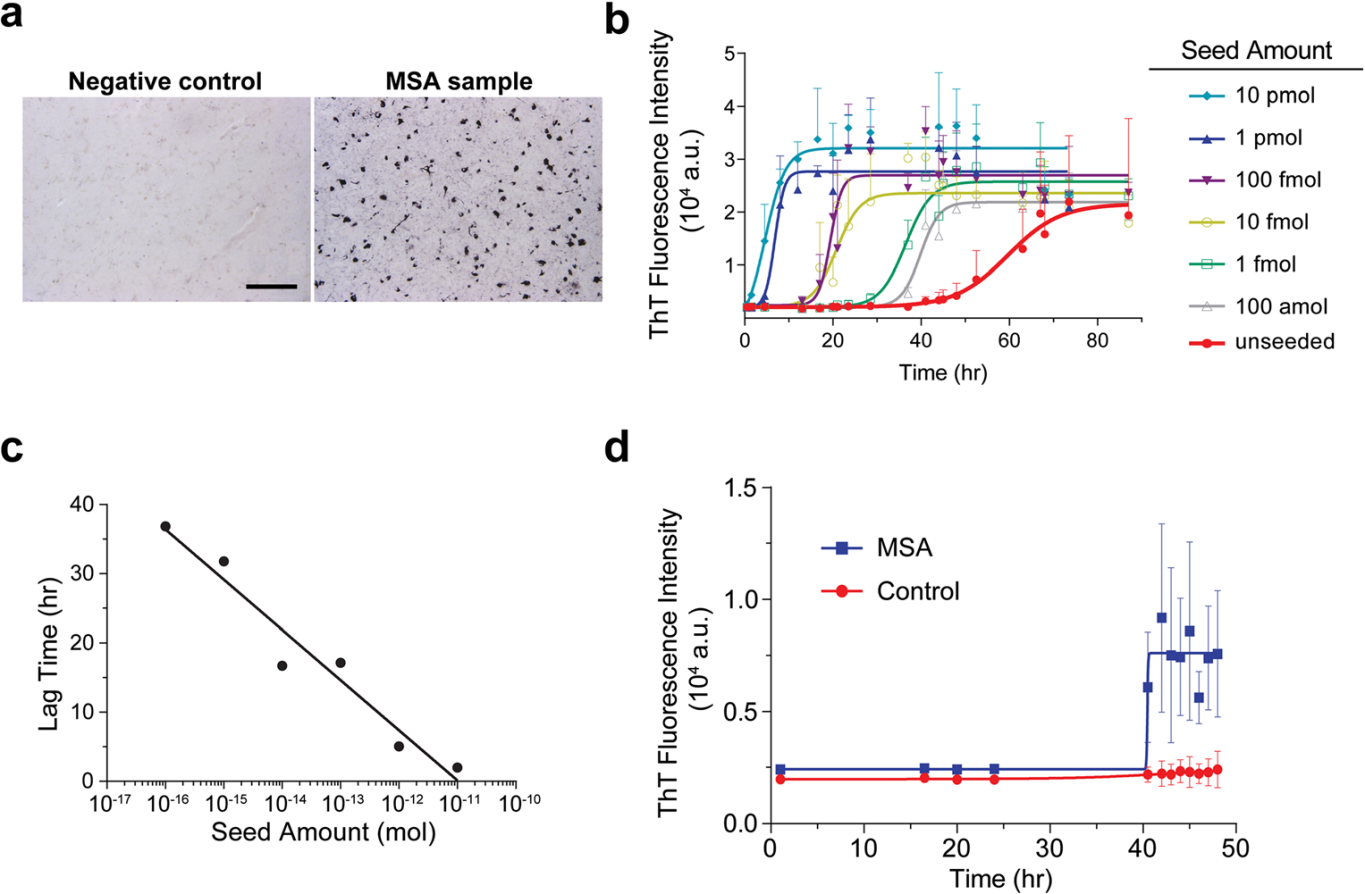
PMCA seeded with fixed human brain samples. **a** Immunohistochemical staining for ps129-α-syn in the striatum of a negative control or a MSA patient as indicated, scale bar = 100 μM. **b** Growth kinetics of α-syn PMCA reactions seeded with indicated amounts of PFFs in the presence of 1% homogenate prepared from formaldehyde-fixed striatum of the control patient. **c** Average lag times of seeded reactions were plotted as a function of the logarithm of the seed amount added to the reaction. Data was fitted with a semilog line. **d** Growth kinetics of α-syn PMCA reactions seeded with 1% homogenate prepared from formaldehyde-fixed striatum of either the control patient (red) or the MSA patient (blue). For **b** and **d**, fibril assembly was monitored by ThT fluorescence intensity at 480 nm, with excitation of 440 nm. Data points in **b** represent the mean ± SD of six replicates. Figure 6d is a representative graph of three independent experiments; each point represents the mean ± SD of four replicates in this experiment. Data was fitted with a sigmoidal curve using the GraphPad Prism software

We first performed immunohistochemical (IHC) staining of a control human striatum sample and confirmed that it did not contain any α-syn aggregates (Fig. 6a, left panel). Adjacent sections were collected and homogenized in 10 volumes of PBS (weight/volume). The presence of 1% final concentration of fixed human brain homogenate in PMCA delayed α-syn fibril growth by about 24 h, increasing the lag time to around 51.5 h. Nevertheless, the assay was still capable of detecting PFFs to a concentration of 100 amol (Fig. 6b), which is similar to the sensitivity of PMCA without any biological tissue (Fig. 2a). Moreover, the lag phases of the seeded PMCA reactions remained quantitative (Fig. 6c).

We then tested fixed tissue from the striatum of a patient with MSA. IHC staining verified the presence of abundant α-syn aggregates in the striatum (Fig. 6a, right panel), which is consistent with the fact that MSA is a neurodegenerative disease characterized by the presence of glial cytoplasmic inclusions of aggregated α-syn [48]. Adjacent sections were used to prepare homogenates. Immunoblot analysis of homogenates prepared from MSA tissue and the control tissue revealed a similar level of total α-syn (Online Resource Fig. S6). When both homogenates were used to seed PMCA, the reaction seeded by MSA homogenate showed a clear ThT signal at about 40 h (Fig. 6d), whereas the reaction seeded by the control homogenate remained at baseline. Based on the calculated lag phase (Fig. 6c), we extrapolated that the amount of seeding-competent α-syn aggregates was around 28 amol. This result revealed that our protocol was able to detect the seeding ability of α-syn aggregate in formaldehyde-fixed tissues and that the fixation was not able to completely eliminate the seeding ability of α-syn aggregates, at least for α-syn aggregates in MSA.

## Discussion

In this study, we optimized the PMCA reaction to detect α-syn aggregates with high sensitivity. The α-syn fibrils created via this process are able to act as seed, recruiting and converting recombinant α-syn in vitro and endogenous α-syn in primary neurons. During the optimization process, we also found that changing PMCA conditions could drastically alter the α-syn fibrilization in both seeded and unseeded reactions. We showed that formaldehyde fixation is not able to completely eliminate the seeding ability of α-syn aggregates, demonstrating the strong seeding activity of α-syn aggregates in patients’ brains.

This is the first report to biochemically detect the seeding activity of α-syn aggregates in formaldehyde-fixed patient brain samples, which demonstrates the sensitivity of our assay and also reveals the powerful seeding capability/stability of α-syn aggregates. This finding is consistent with previous results showing that formaldehyde-fixed prion-diseased brains retain prion infectivity and can be detected by RT-QuIC assay [49]. Similarly, the seeding ability of Aβ in fixed human brain samples has been detected by inoculating the extracts into APP23 transgenic mice [50], and, very recently, the seeding ability of tau aggregates in fixed patients’ brains has been shown using a cell assay [51]. For α-syn, Schweighauser et al. have shown that formaldehyde-fixed tissue from aged Thy1-hA53Tα-syn transgenic mice retains seeding ability when inoculated into young transgenic mice [47], but it is unclear if this is true for a real human patient sample. Moreover, although detecting seeding activity by inoculating transgenic mice is highly sensitive, it leaves open the possibility that instead of seeding, other mechanisms may trigger α-syn aggregation; for example, diseased homogenates may trigger inflammation or innate immune responses, which may subsequently lead to the accumulation of α-syn aggregates. Our biochemical assay provided the direct evidence of seeding activity in fixed tissue, supporting the conclusion that fixation is not able to completely abolish the seeding activity.

Our quantitative estimation reveals that the seeding activity from fixed tissue is rather low, possibly due to several reasons. We chose to use adjacent sections to increase the likelihood that α-syn aggregates were present in the tissue for analysis, but it was impossible for us to know how much aggregate was actually present. For the particular MSA case that we used, there were abundant α-syn aggregates in the striatum, which increased the likelihood for the aggregates to be present in the adjacent sections. However, further modifications to the protocol may ensure that seed is present in the sample, for example, by extracting seed directly from thin paraffin sections [49]. In addition, as recently suggested [51], using sonication may improve the extraction of seeding-competent aggregates. Another factor that may influence the seeding ability is the extent of fixation. In the transgenic mice study [47], the tissue was fixed with formaldehyde for only 48 h. The human tissue used in this study was fixed with formaldehyde for 5 days and stored in cryopreservation buffer for a long time. Longer fixation increases the extent of protein crosslinking, which may reduce α-syn seeding capability. Nonetheless, this is the first time α-syn seeding activity in formaldehyde-fixed patient’s brain has been detected biochemically, which provides strong evidence supporting the “prion-like” spread hypothesis and demonstrates the high sensitivity of our PMCA assay.

The ability of our PMCA protocol to detect femtomole and attomole amounts of α-syn aggregates in mouse and human brain tissues suggest it could be used in a wide variety of applications. Adaptation of this assay to easily accessible tissues or body fluids, such as the CSF, blood, and saliva, could greatly enhance our ability to diagnose and to monitor the progression of synucleinopathies. The quantitative aspect of the assay will be very useful in tracking disease progression and the therapeutic efficacy of potential therapies. In addition, this assay can be used in experimental animal models to monitor the development of seeding-competent α-syn aggregates and correlate it to pathogenic changes, which will be valuable for testing the “prion-like” hypothesis. Our ability to detect the α-syn seeding activity in fixed tissues may also help in testing archived pathological samples and comparing the α-syn seeding activity in different subtypes of synucleinopathies.

## Electronic supplementary material

ESM 1 (DOCX 4996 kb)
