# Supplementary material for: Detecting Alpha Synuclein Seeding Activity in Formaldehyde-Fixed MSA Patient Tissue by PMCA
Source: Mol Neurobiol. 2018 Mar 27;55(11):8728–37. doi: 10.1007/s12035-018-1007-y (PMC6153717; doi:10.1007/s12035-018-1007-y)
Supplement: Supplementary file 1 — (DOCX 4996 kb) [file 12035_2018_1007_MOESM1_ESM.docx]

**Supplemental Materials**

Detecting alpha synuclein seeding activity in formaldehyde-fixed MSA patient tissue by PMCA

Molecular Neurobiology

**Katelyn Becker^1^, Xinhe Wang^1^, Kayla Vander Stel^1^, Toni Divic^1^, Yaping Chu^2^, Jeffrey Kordower^1,2^,**

**Jiyan Ma^1^***

1. Center for Neurodegenerative Science, Van Andel Research Institute, 333 Bostwick Avenue N.E., Grand Rapids, MI 49503, USA

2. Department of Neurological Sciences, Rush University Medical Center, Chicago, IL 60612, USA

* Correspondence should be addressed to: [Jiyan.Ma@vai.org](mailto:Jiyan.Ma@vai.org)

**
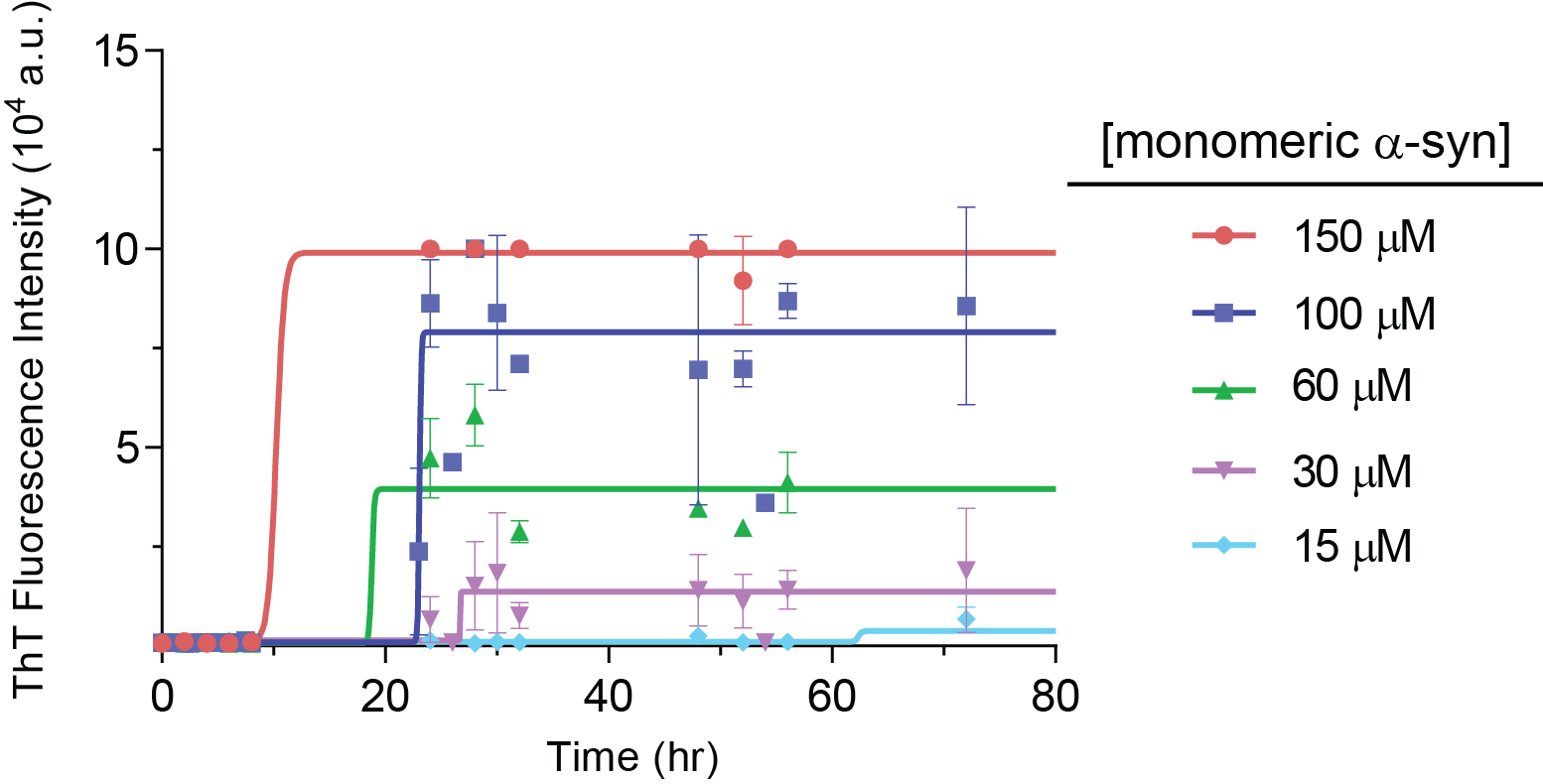
**

**Supplemental Figure S1** **PMCA with different concentrations of α-syn.** PMCA reactions were carried out with standard PMCA settings. The only variation among reactions was the monomeric α-syn substrate concentration as indicated. Each point represents the mean ± SD of 2-8 replicates. Fluorescence measurement and data fitting were as stated in the Fig. 1 caption

**
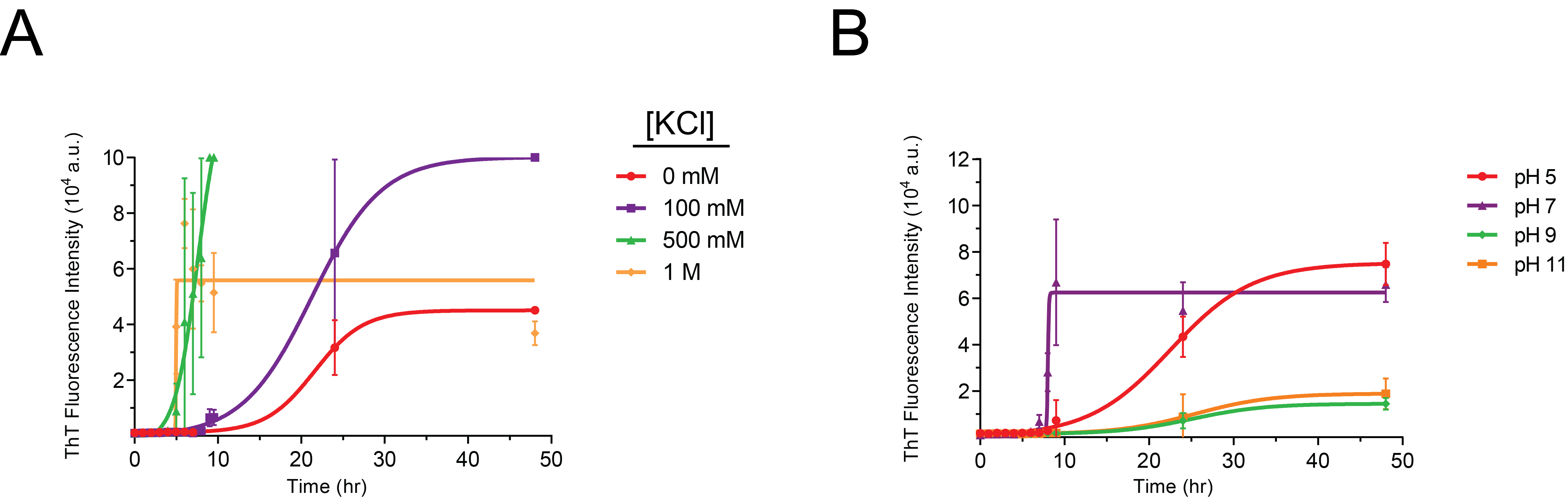
**

**Supplemental Figure S2** **Testing additional conditions with unseeded PMCA reactions.** Unseeded 100 μM α-syn PMCA reactions were carried out with various concentrations of potassium chloride (**A**) or different pH values (**B**). Each point represents the mean ± SD of 3 replicates. Fluorescence measurement and data fitting were as stated in the Fig. 1 caption


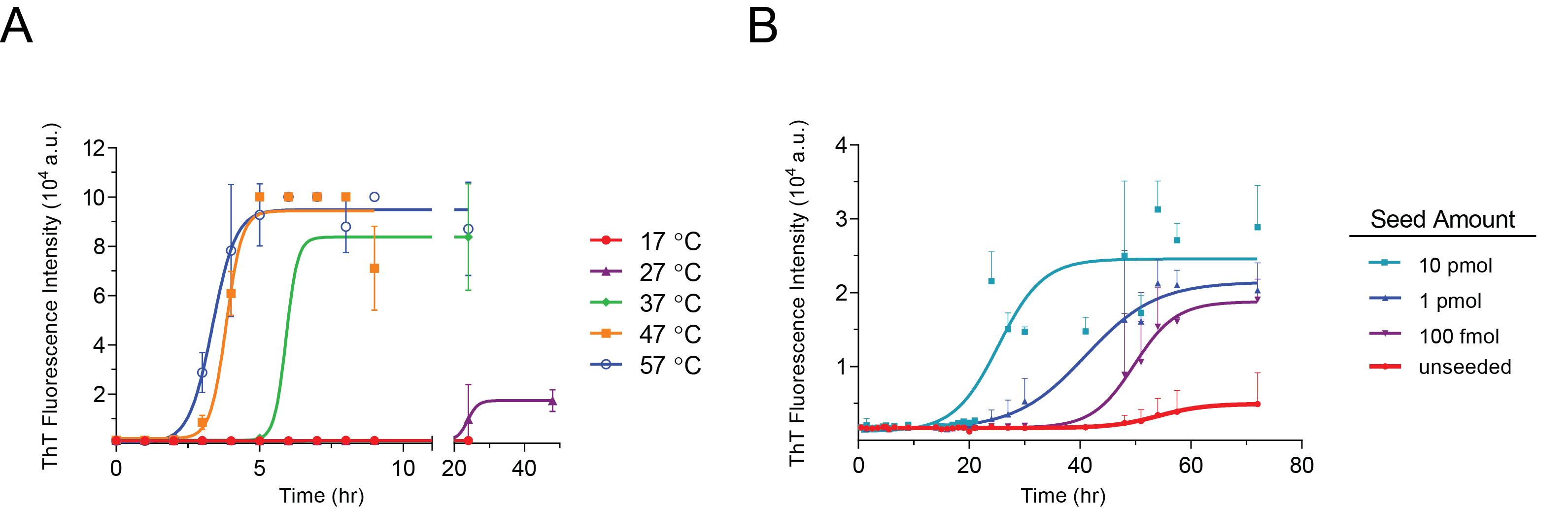


**Supplemental Figure S3 The influence of temperature on PMCA.** Growth kinetics of 100 μM unseeded monomeric α-syn with changes in temperature (**A**), and seeded growth of 50 μM monomeric α-syn as substrate at 27 °C (**B**). Each point represents the mean ± SD of 3 (**A**) or 5 (**B**) replicates. Fluorescence measurement and data fitting were as stated in the Fig. 1 caption


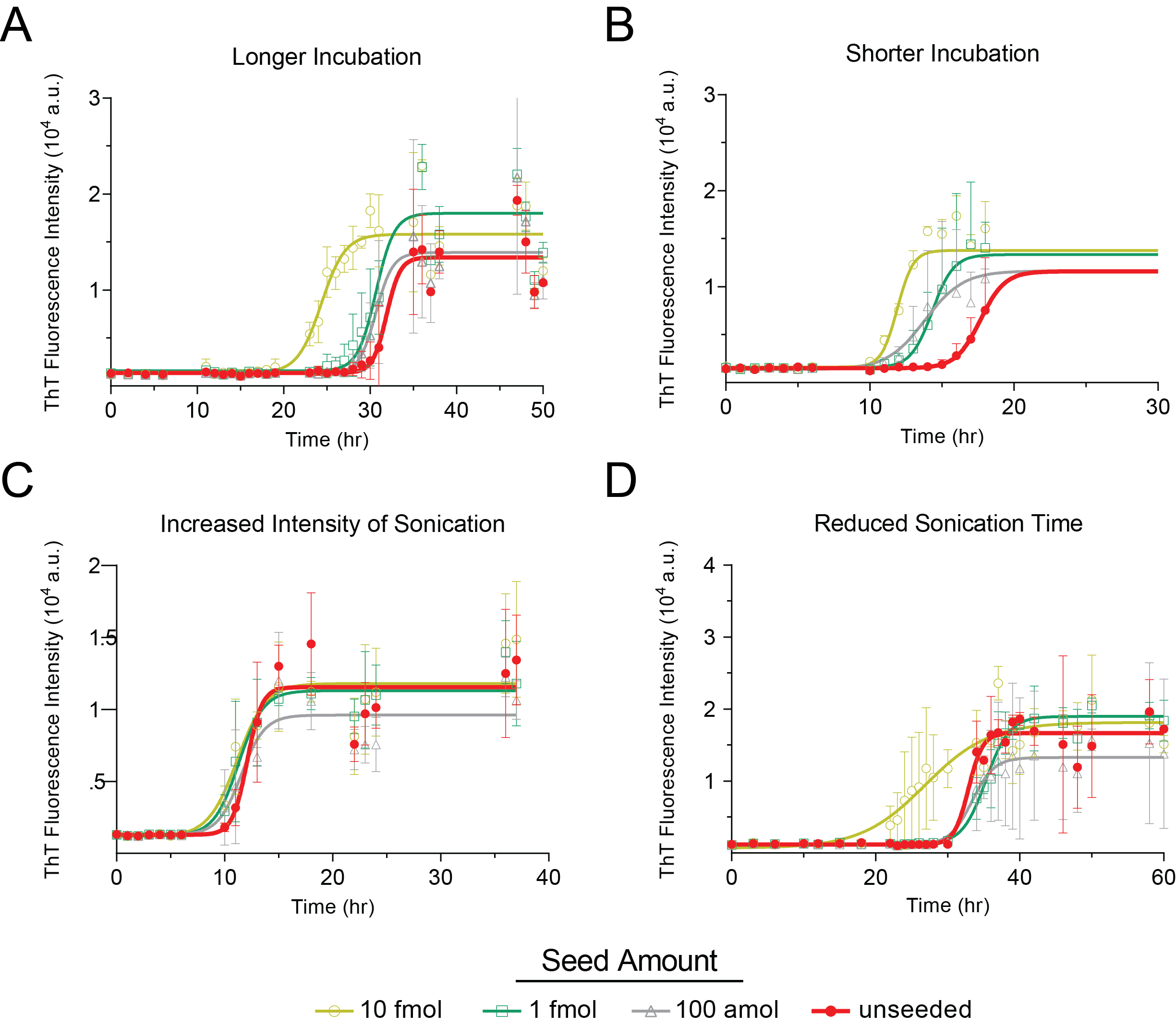


**Supplemental Figure S4** **The influence of sonication on seeded and unseeded PMCA reactions.** PMCA was carried out with longer (**A**) or shorter (**B**) incubation, or with increased sonication intensity (**C**) or reduced sonication time (**D**). Each point represents the mean ± SD of 6 (**A**, **B**, and **C**) or 4 (**D**) replicates. Fluorescence measurement and data fitting were as stated in the Fig. 1 caption

**
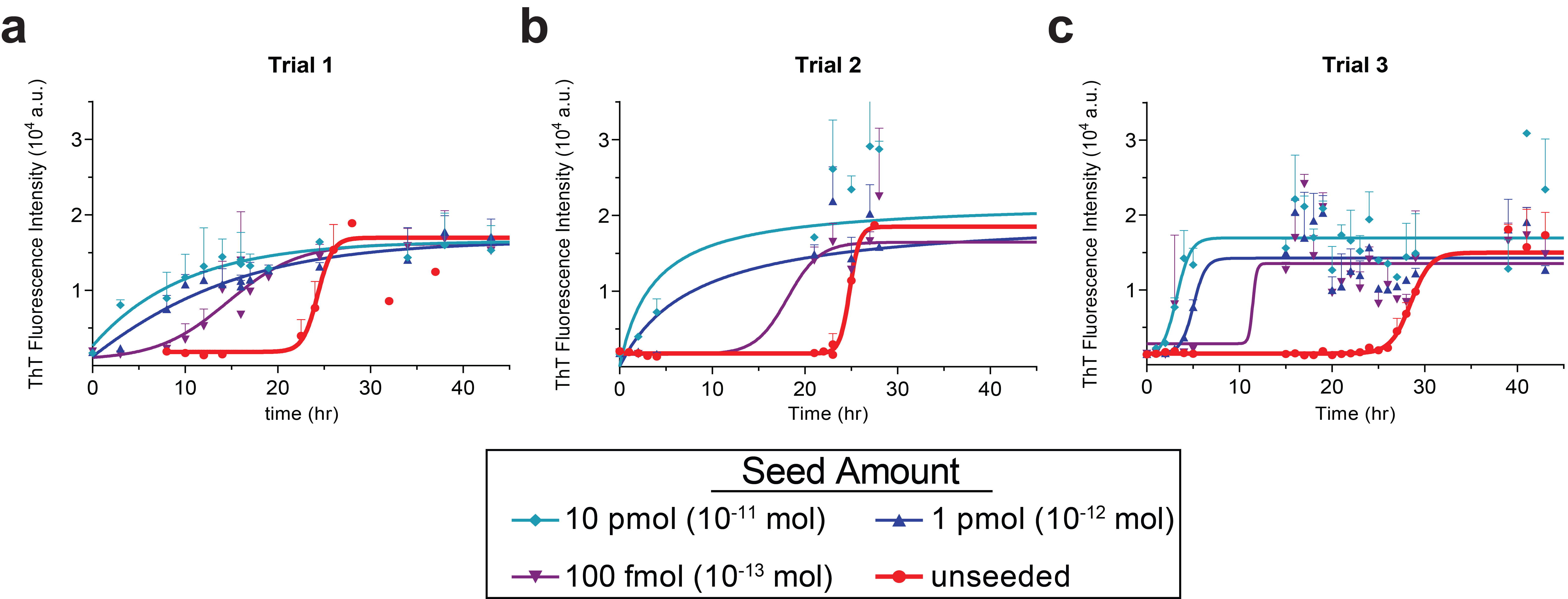
**

**Supplemental Figure S5** **Consistency between seeded and unseeded PMCA experiments.** PMCA experiments (a, b, and c) were carried out one three different days, weeks apart. Each point represents the mean ± SD of 2 (a and b) or 4 (c) replicates. Fluorescence measurement and data fitting were as stated in the Fig. 1 caption

**
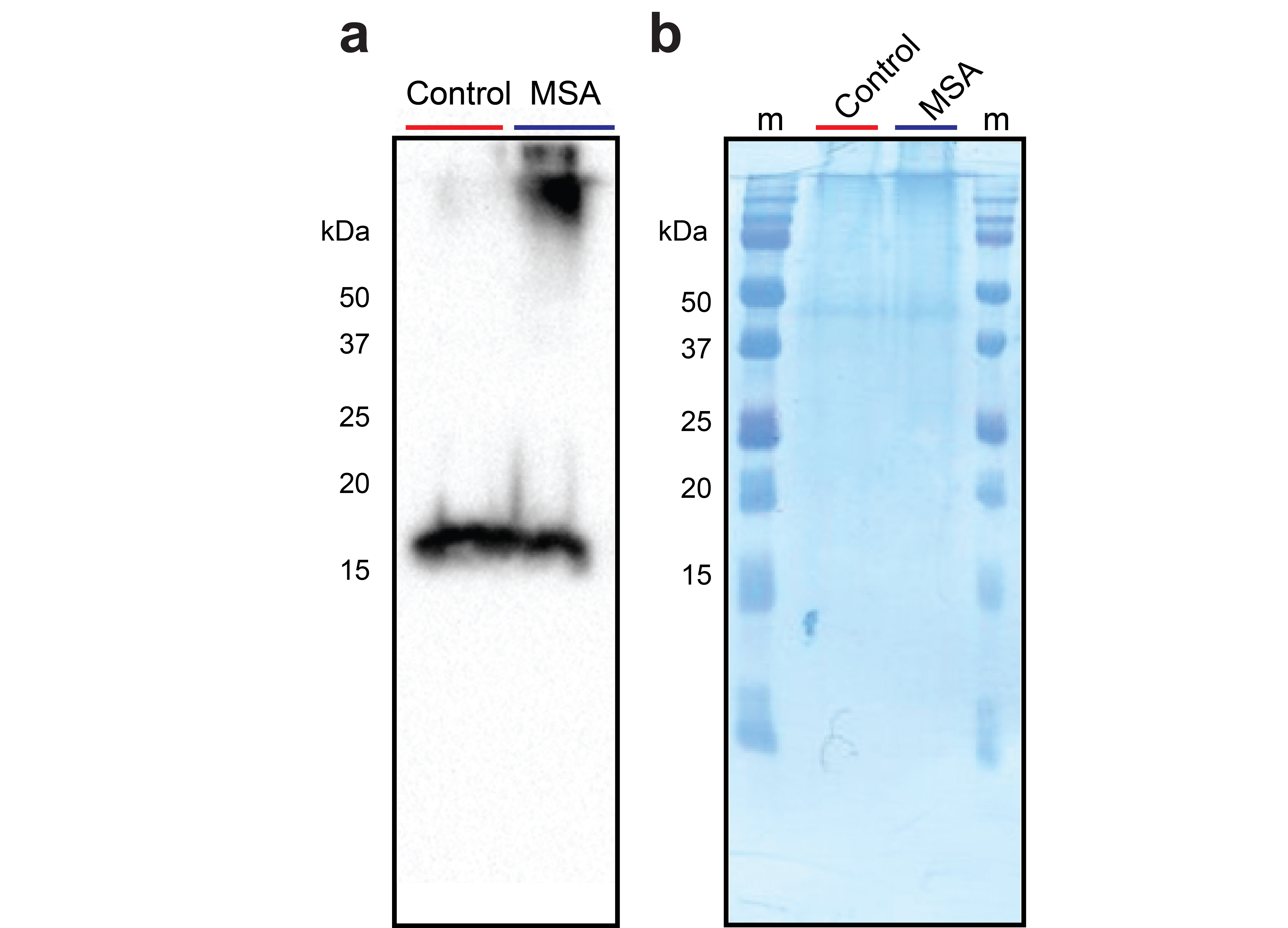
**

**Supplemental Figure S6 Total α-syn in Fixed Brain tissue samples.** Normalized amounts of fixed brain homogenate from either the control (red) patient or MSA (blue) patient were separated by SDS-page and transferred onto a PVDF blotting membrane (GE Healthcare Life Sciences) overnight. (a) The blot was detected with an anti-α-syn monocolonal antibody (Syn211, ThermoFisher Scientific) as the primary antibodies, with Goat anti mouse IgM HRP (Colley) as the secondary antibodies. (b) The amount of protein on the blot was stained by Coomassie blue stain of the membrane.

Since these were paraformaldehyde fixed tissues, most proteins were cross-linked, which prevented them from entering the separating gel (Fig. S6b). The majority of α-syn however, migrated as the monomeric form in SDS-PAGE (Fig. S6a). Some α-syn from MSA sample, very few from the control sample, remained at the border of the separating gel. Overall, the amount of total α-syn is similar in the control and MSA samples.

**Supplemental Table** Statistical differences among reactions seeded by various amounts of PFFs

|  | | Seed amount (mol) | | | | | | |
| --- | --- | --- | --- | --- | --- | --- | --- | --- |
|  |  | 10^-11^ | 10^-12^ | 10^-13^ | 10^-14^ | 10^-15^ | 10^-16^ | 0 |
| Seed amount (mol) | 10^-11^ |  | * | **** | **** | **** | **** | **** |
|  | 10^-12^ | * |  | ** | **** | **** | **** | **** |
|  | 10^-13^ | **** | ** |  | * | **** | **** | **** |
|  | 10^-14^ | **** | **** | * |  | * | **** | **** |
|  | 10^-15^ | **** | **** | **** | * |  | * | **** |
|  | 10^-16^ | **** | **** | **** | **** | * |  | *** |
|  | 0 | **** | **** | **** | **** | **** | *** |  |

The lag phases from seeded PMCA reaction (Fig. 2) were calculated as the ThT reading 3X that of the baseline reading for each of the 12 replicates, and statistical analysis was carried out using a one-way ANOVA. p value symbols are as follows: * ≤ 0.05, ** ≤ 0.01, *** ≤ 0.001, **** ≤ 0.0001
